# A Systematic Review of Studies of Attitudes and Beliefs of Healthcare Professionals Towards Non-Epileptic Attack Disorder (NEAD)

**DOI:** 10.1192/bjo.2024.263

**Published:** 2024-08-01

**Authors:** Amelia Townsend, James Dobrzanski, Sukhi Shergill, Joanne Rodda

**Affiliations:** 1 Kent and Medway Medical School, Canterbury, United Kingdom; 2 Kent and Medway NHS and Social Care Partnership Trust, Canterbury, United Kingdom; 3 Kings College London, London, United Kingdom

## Abstract

**Aims:**

Non epileptic attacks (also referred to as psychogenic non-epileptic seizures, functional seizures or dissociative seizures) are similar in appearance to epileptic seizures but are not accompanied by ictal electroencephalographic (EEG) discharges. NEAD is classified as either a conversion or dissociative disorder in DSM-V and ICD11 respectively, and is often associated with significant long-term disability. People with NEAD often access care across many different specialties and healthcare settings. Their experiences of doing so are frequently negative, based both on interactions with clinicians and integration of care.

The aims of this study were to review the existing literature on the attitudes of clinicians towards non-epileptic attack disorder (NEAD), and any differences that exist between professional groups.

**Methods:**

The study followed PRISMA 2020 guidelines and was registered on the international prospective register of systematic reviews (PROSPERO). Three electronic databases (MEDLINE, EMBASE and PsycInfo) were searched for studies of clinician attitudes towards NEAD using pre-developed terms. These terms were optimised following familiarisation with the literature. Specific inclusion and exclusion criteria were applied, and studies were selected if they included data regarding the attitudes of healthcare professionals from any group towards NEAD. A data extraction template was used to synthesise study characteristics and outcomes. The Mixed Methods Appraisal Tool was used to appraise methodological quality of the included studies. Two reviewers independently completed the selection process and data extraction.

**Results:**

The search strategy yielded 2885 citations, of which 76 were selected for review of the full publication based on the title and abstract. Inclusion/exclusion criteria were applied to full texts. The literature mainly included clinicians from general practice, neurology, emergency department and psychiatry. There was general negative stereotyping of people with NEAD and a lack of confidence in management. Attitudes differed between professions, particularly with respect to aetiology.

**Conclusion:**

The literature highlighted that many clinicians held a negative attitude towards people with NEAD, and there was evidence of a general lack in confidence towards NEAD across all healthcare professional groups. There was a difference between healthcare professional groups, mostly related to views on aetiology. The review highlights the need for greater education related to NEAD with a focus on understanding aetiology and greater transparency in interdisciplinary working.

